# Association of *FTO* gene variant rs9939609 with polycystic ovary syndrome from Gujarat, India

**DOI:** 10.1186/s12920-023-01654-0

**Published:** 2023-09-14

**Authors:** Hiral Chaudhary, Jalpa Patel, Nayan K. Jain, Sonal Panchal, Naresh Laddha, Rushikesh Joshi

**Affiliations:** 1 Department of Biochemistry and Forensic Science, University School of Sciences, Gujarat University, Ahmedabad, 380009 Gujarat India; 2 Department of Life Science, University School of Sciences, Gujarat University, Ahmedabad, 380009 Gujarat India; 3 Dr. Nagori’s Institute for Infertility and IVF, Ahmedabad, Gujarat India; 4 In Vitro Specialty Lab Pvt Ltd, Navrangpura, Ahmedabad, 380009 Gujarat India

**Keywords:** PCOS, *FTO* gene, Single nucleotide polymorphism, Obesity, Insulin resistance

## Abstract

**Background:**

Polycystic ovary syndrome is a multifactorial endocrine disorder impacting women of reproductive age. Variations within the *FTO* gene have been linked to both obesity and type 2 diabetes mellitus. Given that PCOS is frequently associated with obesity and compromised glucose tolerance, we investigated the prevalence of the rs9939609 variant within the *FTO* gene among women diagnosed with PCOS and a control group. Our aim is to uncover potential correlations between this genetic variant, metabolic attributes, and endocrine markers within the Gujarat province of India.

**Method:**

We enrolled a total of 114 participants, (62 individuals diagnosed with PCOS and 52 healthy controls). DNA extraction from venous blood was conducted for all participants. The rs9939609 polymorphism was investigated through tetra-primer amplification refractory mutation system-polymerase chain reaction. Furthermore, we performed biochemical assessments to quantify levels of estradiol, luteinizing hormone (LH), follicle-stimulating hormone (FSH), thyroid-stimulating hormone (TSH), total testosterone, prolactin (PRL), and Dehydroepiandrosterone sulfate (DHEAS). Statistical analyses were carried out utilizing SPSS version 21 (IBM, USA).

**Results:**

The present study did not reveal any noteworthy association between cases and controls. The frequencies of genotypes and alleles within the cohorts displayed no statistically significant differences (*p* = 0.25, *p* = 0.68, and *p* = 0.78, respectively). The dominant model indicated a modest risk (OR:1.13, 95%CI: 0.55 to 2.38) toward PCOS development. There was a noticeable statistical difference observed in the levels of total testosterone, DHEAS, and BMI between the case and control groups (*p* < 0.002, *p* < 0.0002, *p* < 0.0008). However, no variations in clinical variables were observed among genotypes within the PCOS group.

**Conclusion:**

This is the first study to investigate the association of *FTO* gene polymorphism and PCOS in Gujarati population. Our study findings indicate that the *FTO* gene variant is not directly linked to the onset of PCOS. However, it appears to exert an influence on metabolic factors such as obesity and insulin resistance. Notably, our results suggest that insulin resistance is more frequently observed among PCOS patients who are obese, as compared to those with non-obese PCOS patients.

**Supplementary Information:**

The online version contains supplementary material available at 10.1186/s12920-023-01654-0.

## Introduction

Polycystic ovary syndrome (PCOS) is the most common endocrinopathy affecting women of reproductive age, characterized by hyperandrogenism, menstrual irregularities, and polycystic ovarian morphology [1]. PCOS affects 2.2% to 26% of the global population, with India having a prevalence rate of 11.96% when diagnosed by Rotterdam criteria [2–4]. Women diagnosed with PCOS commonly experience disruptions in their hormonal balance, insulin resistance (IR), and metabolic functions. These issues can subsequently lead to the development of fertility issues (FI), type 2 diabetes mellitus (T2DM), and cardiovascular disease (CVD), collectively affecting the woman’s overall quality of life [5]. Although the etiology of PCOS is unclear, the most common co-morbidities are obesity and diabetes mellitus [6]. Research shows that roughly 50% of PCOS women are overweight or obese, implying that obesity plays a significant role in the disease’s pathophysiology. Obesity and PCOS have a strong inherited basis, indicating a shared genetic predisposition contributing to their co-occurrence [7, 8].

Researchers have explored PCOS extensively over time, suggesting various hypotheses about its origins and distinct characteristics. Nonetheless, the exact cause of the syndrome remains uncertain. Genetic studies have identified around 100 susceptibility genes linked to PCOS. Unlike candidate gene methods focused on smaller samples, genome-wide association studies (GWAS) provide a systematic and unbiased means to investigate numerous genome-wide variations in both affected individuals and healthy controls. This approach aids in revealing connections between genetic variants and complex conditions like PCOS [9]. The identification of the fat mass and obesity-associated gene (*FTO*) marked a significant milestone as it became the first gene with substantial influence on an individual’s susceptibility to common polygenic obesity. The human *FTO* gene is located on chromosome 16q12.2 within the first intron and is widely expressed across various tissues, including adipose tissue, suggesting a potential role in regulating body weight [10, 11]. This gene encodes a protein belonging to the nonheme dioxygenase superfamily (Fe(II)- and 2-oxoglutarate-dependent dioxygenases), participating in a range of cellular processes [12, 13]. Given that PCOS, particularly in its well-defined form, often manifests during adolescence, particularly among South Asian populations, the *FTO* variants exhibit associations with insulin resistance and glucose intolerance in PCOS [14]. While research has explored the connection between *FTO* variants and insulin-related factors, there remains a gap in the literature regarding the link between *FTO* variants and hyperandrogenaemia [15].

Recent studies have shown that PCOS women have a higher risk of obesity and type 2 diabetes mellitus due to a common single nucleotide polymorphism (SNP) (rs9939609) of the *FTO* gene with a T to A change [16]. Several studies were conducted to prove the impact of *FTO* variants on the risk of PCOS, but the results were conflicting across ethnic groups [17–21]. More research is needed to speculate the true impact of polymorphisms in the *FTO* gene on PCOS in other populations.

In the present study, we aim to examine whether the prevalence of the *FTO* gene variant (rs9939609) differs between PCOS-affected women and healthy controls from Gujarat and the associations between these genetic factors and metabolic characteristics and endocrine parameters.

## Methods and materials

### Sample collection

The study was conducted at Gujarat University’s Department of Biochemistry and Forensic Science, Ahmedabad, Gujarat. There were 62 PCOS patients aged 12 to 40 and 52 healthy control women in the same age range (Supplementary Fig. 1). The entire patient population was recruited from Dr. Nagori’s Institute for Fertility and IVF Hospital in Ahmedabad, Gujarat, between June 2021 and December 2022. They were diagnosed with PCOS based on the Rotterdam Revised 2003 criteria (2 out of 3). 1) oligomenorrhea or amenorrhea for at least 6 months; 2) clinical or biochemical signs of hyperandrogenism; and 3) polycystic ovaries (the presence of 12 or more follicles in each ovary measuring 2-9 mm in diameter), as well as the exclusion of congenital adrenal hyperplasia, Cushing’s syndrome, androgen-secreting tumor, hyperprolactinemia, and thyroid dysfunction. They had not taken hypoglycemic medications or hormonal therapy, including oral contraceptives, for at least three months. All methods were carried out in accordance with relevant guidelines and regulations, also the study was approved by Gujarat University’s Institutional Ethical Committee (Reference number GU-IEC(NIV)/02/Ph.D./006), and all study participants provided written informed consent.

The PCOS group was further divided into obese patients with a BMI of ≥ 30 (*n* = 41) and non-obese patients with a BMI of < 25 (*n* = 21) [22, 23]. The Homeostatic Model Assessment of Insulin Resistance (HOMA-IR) is an easy and non-invasive way to figure out how sensitive your body is to insulin [24]. The PCOS patients were classified as insulin resistant (HOMA IR ≥ 2.0, *n* = 35) or non-insulin resistant (HOMA IR < 1.9, *n* = 27) using the Homeostasis Model Assessment of Insulin Resistance (HOMA IR) [25]. If the value is greater than 2.0, it suggests that a person’s body might be resistant to insulin. Each subject in this study completed a clinical proforma, which detailed the patient’s condition regarding menstrual history, infertility, the onset, and severity of PCOS clinical symptoms, drug use history, and diabetes family history. BMI was calculated by dividing weight in kilograms by height in centimetres. Obesity was defined as having a BMI greater than 30 kg/m2 [23].

### Biochemical analysis

On the second and third days of the menstrual cycle, five millilitres of venous blood were drawn from each subject, and the serum was then separated. The routine biochemical analysis included measurements of prolactin, estradiol, luteinizing hormone (LH), follicle-stimulating hormone (FSH), thyroid-stimulating hormone (TSH), total testosterone, and Dehydroepiandrosterone Sulfate (DHEAS). These hormones were quantified using the commercially available Chemiluminescent immunoassay (CLIA) kits (Siemens centaur CP.,Siemens Diagnostics, Germany) at In Vitro Specialty Lab Pvt Ltd, Navrangpura, Ahmedabad, Gujarat, India. The HOMA IR is calculated as [Glucose (mg/dL) x [Insulin (U/mL) / 405.

### DNA Extraction

The genomic DNA of all study participants was extracted from 0.5 M EDTA mixed with 2 millilitres of peripheral blood using the standard phenol–chloroform method with minor modifications [26]. Using a Nanodrop, the purity and concentration of DNA were determined, and the DNA samples were stored at -80ºC until further analysis.

### Selection of study of candidate gene and SNP of PCOS

Ensembl, SNPedia, and CinVar were used to select SNP for this study. The goal was to find an SNP that has been linked to high PCOS susceptibility in Asian populations. PCOS is frequently associated with obesity, hyperinsulinemia, type 2 diabetes mellitus, hypertension, dyslipidemia, and cardiovascular disorders. Given the high incidence of obesity and type 2 diabetes in this ethnic group, we chose to investigate polymorphisms in the *FTO* gene, specifically rs9939609, which has been linked to obesity.

### Genotyping

**Fig. 1:**
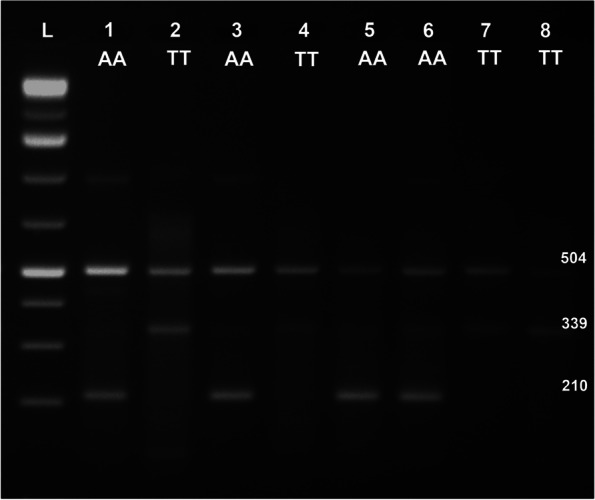
Genotyping of *FTO* variant rs9939609 through tetra-ARMS PCR. Genotypes were distinguished by a 210-bp band for the A allele, a 339-bp band for the T allele, and a 504-bp common band indicating wild-type homozygous (TT), polymorphic homozygous (AA) and heterozygous (AT)

*FTO* SNP rs9939609 T > A was genotyped directly using appropriate primers through tetra-Amplification refractory mutation system polymerase chain reaction (ARMS PCR). Amplification for *FTO* being achieved using outer forward primers OF: 5’- AGGAGAGGAGAAAGTGAGCT -3’ and outer reverse primers OR: 5’- TGTTCAAGTCACACTCAGCCTC -3’ and inner forward primers IF: 5’- CCTTGCGACTGCTGTGAATTTA -3’ and inner reverse primers IR: 5’- CAGAGACTATCCAAGTGCATCACA -3’ (Eurofins Scientific, Bangalore, India). In brief, tetra-ARMS PCR was performed on each sample in a 20ul PCR reaction mixture containing 50 ng of genomic DNA, 10ul of DreamTaq Green PCR Master Mix (2X) (Thermofisher scientific- USA), and 0.4ul of each primer (10 pmol/L) filled with PCR-grade water. The thermal cycler was used for the PCR, which included an initial denaturation at 95°C for 10 min, followed by 35 cycles of denaturation at 95°C for 30 s, annealing at 58°C for 45 s, extension at 72°C for 45 s, and final extension at 72°C for 10 min. All PCR amplicons were visualized under UV light using 2% agarose gel electrophoresis stained with ethidium bromide. As shown in Fig. 1, genotypes were distinguished by a 210-bp band for the A allele, a 339-bp band for the T allele, and a 504-bp common band.

### Statistical analysis

To determine the significance of clinical and biochemical characteristics in the prognosis of polycystic ovary syndrome and their impact on disease development, statistical analysis was performed using SPSS (Statistical Package for social science) for Windows version 21 (IBM, USA) to determine the *p*-value. The results are presented as mean ± standard deviation (SD) for normally distributed variables. The student t-test was used to compare continuous measures between cases and controls. The chi-square test was used to compare categorical variables and assess genotype and allele frequency deviation from Hardy–Weinberg equilibrium. The odds ratios (ORs) and confidence intervals (95% CIs) were computed. BMI, testosterone, luteinizing hormone, and other clinical variables were compared using a one-way analysis of variance (ANOVA). Patients were divided into two groups for each polymorphism studied: one group included heterozygous and risk homozygous subjects, and the second group included non-risk homozygous subjects. We combined TA (heterozygous) and AA (polymorphic homozygous) as one group and wild-type TT (wild homozygous) as the second group using a dominant model. To assess the extent of the risk factors that contribute to the onset of PCOS, we first compared all cases and controls by calculating the crude odds ratio (OR) using the binary logistic regression method. A significant association was at 95% significance levels of *p* < 0.05.

## Results

In the study subjects, the overall mean ± SD age of cases and controls was 30 ± 4 and 26 ± 7. The mean ± SD for BMI calculated was 27.23 ± 5.64 and 23.63 ± 5.31 in cases and controls. Age and BMI differed statistically between cases and controls (*p* < 0.05). Furthermore, the total testosterone and DHEAS levels were statistically significant between the groups (*p* < 0.05) (Table 1).

**Table 1:** Comparison of demographic and biochemical features between PCOS and controls

**Clinical characteristics**	**Cases (*n* = 62)**	**Control (*n* = 52)**	***p* Value**
	**Mean ± SD**	**Mean ± SD**	
Age	30 ± 4	26 ± 6	0.0001^*^
BMI	27.23 ± 5.64	23.63 ± 5.31	0.0008^*^
TSH	2.22 ± 1.1	2.15 ± 1.32	0.7560
LH	6.74 ± 4.43	5.35 ± 4.37	0.0900
FSH	8.89 ± 7.44	7.99 ± 6.48	0.5000
LH/FSH	1.00 ± 0.93	0.94 ± 1.06	0.7410
E2	80.2 ± 111.49	75.32 ± 25.36	0.7500
TT	23.18 ± 13.97	16.17 ± 8.89	0.002^*^
PRL	15.68 ± 11.47	17.83 ± 22.01	0.5080
DHEAS	126.03 ± 58.2	199.67 ± 136.53	0.0002^*^
F insulin	9.83 ± 4.06	Not checked	-
f glucose	85.89 ± 6.77	Not checked	-
HOMA_IR	2.08 ± 0.87	Not checked	-

*BMI* Body Mass Index, *TSH* Thyroid stimulating hormone, *LH* luteinizing hormone, *FSH* follicle stimulating hormone, *E2* estradiol, *TT* total testosterone, *PRL* prolactin, *DHEAS* dehydroepiandrosterone sulphate, *F. Insulin* fasting insulin, *F. glucose* fasting glucose, *HOMA IR* homeostasis model assessment of insulin resistance

^*^p < 0.05 significant

Table 2 presents the distribution of genotypes between PCOS patients and the control group. The frequencies of rs9939609 genotypes in the *FTO* gene were as follows: 40.38% TT, 30.77% TA, and 28.85% AA for controls, and 43.55% TT, 19.35% TA, and 37.10% AA for cases. The corresponding allele frequencies were 55.77% T allele and 44.23% A allele for controls, and 53.23% T allele and 46.77% A allele for cases.

**Table 2:** Distribution of genotype, allele frequencies and genetic models of rs9939609 between PCOS cases and controls

**SNP**	**Genotype/ Allele**	**Case (%)**	**Control (%)**	***p*-value**	**χ**^**2**^	**OR (95% CI)**
rs9939609	TT	27 (43.55)	21 (40.38)	0.25 0.68	1.27 0.15	1.71
	TA	12 (19.35)	16 (30.77)			0.69 to 4.51
	AA	23 (37.10)	15 (28.85)			0.83
						0.33 to 2.00
	T	33 (53.23)	29 (55.77)	0.78	0.07	0.90
	A	29 (46.77)	23 (44.23)			0.43 to 1.83
Dominant model	T/T	35 (56.45)	31 (59.62)	0.73	0.11	1.13
	T/A + A/A					0.55 to 2.38
Recessive model	T/A + T/T	39 (62.90)	37 (71.15)	0.35	0.86	1.45
	A/A					0.65 to 3.13
Heterozygous model	T/T + A/A	50 (80.65)	36 (69.23)	0.15	1.98	0.54
	T/A					0.23 to 1.27

*χ*^*2*^ chi Square, *OR* odds ratio, *CI* confidence interval

*p* < 0.05 significant

However, the differences in genotypic and allelic frequencies of rs9939609 showed no significant differences between PCOS cases and controls (TT vs TA *p* = 0.25 [OR: 1.71(0.69–4.51)]; TT vs AA *p* = 0.68 [OR: 0.83(0.33–2.00)]; and T vs A *p* = 0.07 [OR: 0.90(0.43–1.83)]. Due to the limited number of A/A genotypes in our study, we grouped T/A and A/A genotypes as a dominant model to assess the link between *FTO* rs9939609 genotypes and PCOS patients. However, the genetic models did not reveal any significant differences between the groups (Table 2). In the context of rs9939609, the Hardy–Weinberg Equilibrium (HWE) was computed and verified exclusively for control subjects. The results of the analysis revealed a genotype distribution with a χ 2 = 7.36 and a *p* = 0.006.

The *FTO* genotype distribution between obese vs. non-obese and insulin resistant vs. non-insulin resistant in PCOS patients was also studied in our population. The genotype analysis showed no significant difference with 1.04- and 1.27-fold risk towards developing obesity and insulin resistance in PCOS patients (Tables 3 and 4). However, we have observed a significant difference between the obese PCOS patients with insulin resistant PCOS patients (χ^2^ = 13.76, OR = 8.72 [95% CI 2.65–26.98] *p*-value < 0.05). In our study, obese PCOS individuals are more prone to insulin resistance than non-obese PCOS patients.

**Table 3:** Comparative analysis of *FTO* genotypes and alleles between obese and non-obese PCOS patients

**SNP**	**Genotype/ Allele**	**Obese (%) *n* = 41**	**Non-Obese (%) *n* = 21**	***p*-value**	**χ**^**2**^	**OR (95% CI)**
rs9939609	TT	18 (43.90)	9 (42.86)	0.701	0.709	-
	AT	9 (21.95)	3 (14.29)			
	AA	14 (34.15)	9 (42.86)			
	T	22 (53.66)	10 (47.62)	0.652	0.202	1.27
	A	19 (46.34)	11 (52.38)			0.46 to 3.62
Dominant model	T/T	23 (56.10)	12 (57.14)	0.937	0.006	1.04
	T/A + A/A					0.37 to 3.09

*χ*^*2*^ chi Square, *OR* odds ratio, *CI* confidence interval

*p* < 0.05 significant

**Table 4:** Comparative analysis of *FTO* genotypes and alleles between insulin-resistant and non-insulin-resistant PCOS patients

**SNP**	**Genotype/ Allele**	**Insulin resistant (%) *n* = 35**	**Non-Insulin resistant (%) *n* = 27**	***p*-value**	**χ**^**2**^	**OR (95% CI)**
rs9939609	TT	16 (45.71)	11 (40.74)	0.871	0.275	-
	AT	7 (20.00)	5 (18.52)			
	AA	12 (30.29)	11 (40.74)			
	T	19 (54.29)	13 (48.15)	0.631	0.229	1.27
	A	16 (45.71)	14 (51.85)			0.44 to 3.26
Dominant model	T/T	19 (54.29)	16 (59.26)	0.695	0.153	1.22
	T/A + A/A					0.42 to 3.22

*χ*^*2*^ chi Square, *OR* odds ratio, *CI* confidence interval

*p* < 0.05 significant

The one-way ANOVA was used to analyse the clinical parameters concerning all the genotypes of rs9939609 variant in women with PCOS. None of the genotypes showed any significant distribution (Table 5). We discovered that PCOS is related to BMI, serum testosterone, and DHEAS concentrations using logistic regression analysis (Supplementary Table 1). BMI and serum testosterone levels remained statistically significant, with a 1.11-fold and 1.13-fold increased risk of developing PCOS, respectively. DHEAS, on the other hand, showed no risk in our study (OR: 0.982; *p* value 0.001). A Hosmer and Lemeshow test value of 0.12 indicated that the model was significant (*p* > 0.05 was considered significant). Furthermore, the model’s efficacy was demonstrated by its ability to explain 47.1% of the variation among PCOS patients, as measured by the Nagelkerke R^2^. Furthermore, the model classified 79.8% of the cases correctly, demonstrating its good fit and accuracy. Interestingly, the model did not show a significant connection between rs9939609 genotypes, LH, FSH, LH/FSH ratio, estradiol, PRL, and PCOS.

**Table 5:** Comparative analysis of different studied parameters in relation to *FTO* genotypes in PCOS patients

**Clinical characteristics**	**rs9939609**			***p*-value**
	**AA**	**AT**	**TT**	
BMI	27.98 ± 6.71	25.45 ± 3.55	27.38 ± 5.22	0.45
TSH	2.02 ± 1.02	2.27 ± 0.9	2.38 ± 1.21	0.52
LH	6.8 ± 4.03	6.93 ± 5.92	6.62 ± 3.95	0.97
FSH	10.43 ± 11.26	6.85 ± 3.53	8.47 ± 3.06	0.38
LH/FSH	1.04 ± 1.09	1.2 ± 1.14	0.88 ± 0.6	0.61
E2	68.44 ± 45.7	123.83 ± 234.44	70.83 ± 35.85	0.33
TT	21.02 ± 11.1	25.29 ± 21.65	24.09 ± 11.34	0.63
PRL	19.8 ± 16.8	13.79 ± 6.74	13.02 ± 4.26	0.09
DHEAS	129.34 ± 68.24	125.34 ± 56.93	123.52 ± 48.53	0.94
HOMA_IR	2.05 ± 0.76	2.07 ± 0.91	2.12 ± 0.93	0.96

One way ANOVA

*p* < 0.05 significant

## Discussion

Polycystic ovary syndrome (PCOS) is a complex condition linked to obesity. The Fat mass and obesity-associated gene (*FTO*) is thought to be closely related to obesity. As a result, *FTO* gene stands out as a promising candidate gene associated with PCOS. The Indian community is transitioning towards fast-food diets, decreasing physical activity, and experiencing elevated levels of adiposity and obesity in urban regions compared to rural ones. The interplay between genetic elements and lifestyle decisions has played a role in the rise of obesity and its related health concerns. Nonetheless, the precise relationship between *FTO* and PCOS is unknown and needs to be clarified, particularly across diverse ethnic groups. Therefore, the present study was designed to investigate the relation of the *FTO* gene variant (rs9939609) with PCOS. In previously reported studies, this polymorphism has been widely studied to evaluate the potential association with PCOS, but the results needed to be more consistent.

BMI is a measure of obesity that sheds light on the issues obesity causes. The *FTO* gene, expressed widely with the highest levels in the hypothalamus, is associated with obesity [10]. In our study, there was a statistically significant difference in Age and BMI (*p* < 0.05) (Table 1). Our findings suggest that the mutant carrier A allele contributes to the development of PCOS by increasing BMI in PCOS patients. In the current study, the BMI of PCOS women was significantly higher than controls which agreed with other studies [7, 20]. Moreover, obesity is a multigenic complex disorder that increases the risk of type 2 diabetes, cardiovascular morbidities, and other health complications such as infertility and poor pregnancy outcomes in PCOS women.

We also conducted a correlation analysis between the obese vs. nonobese PCOS group and the insulin resistant vs. non-insulin PCOS group. There was neither significant difference between obese vs. non-obese PCOS patients nor between insulin resistant vs. non-insulin resistant PCOS patients (Tables 3 and 4). In addition, we have conducted the association between obese and insulin resistant PCOS patients, and there was a significant difference between both groups (*p* < 0.05, OR = 8.75, CI 2.65–26.98). This study’s findings align with other investigations into the connection between obesity and PCOS [23, 27, 28].

The current study evaluated and compared the distribution of genotypic and allelic frequencies between groups. Our study showed that the genotypic and allelic distribution of rs9939609 was not statistically different between cases and controls (*p* values 0.73 and 0.78) (Table 2). The (T/A + A/A) genotypes frequencies were not significantly different in PCOS patients compared to the controls (56.45% vs. 59.62%) vs. T/T genotype (43.55% vs. 40.38%), OR = 1.13 [95% CI 0.55–2.38] (*p* > 0.05) (Table 2). Previous studies conducted in the Chinese, UK, Finland, and South Brazilian populations found a strong correlation between *FTO* and PCOS [17–19, 29]. In contrast, others found a link between *FTO* and BMI in PCOS women, though they do not appear to play a significant role in the reproductive phenotypes of PCOS [21, 30]. A meta-analysis by Cai et al. found that the *FTO* rs9939609 polymorphism was linked with PCOS risk among East Asians but not in the Caucasian population [31].

We found a significant association between Total Testosterone and DHEAS in the PCOS group compared to the control group. Levels of Total testosterone (*p* < 0.05) were higher, whereas DHEAS levels (*p* < 0.05) were low in PCOS patients as compared to healthy controls (Table 1), which was consistent with the study conducted by Wehr et al. [7, 32]. Furthermore, in our research, LH, FSH, PRL, estradiol, total testosterone and DHEAS levels were also analyzed concerning genotypes of the *FTO* gene. No significant difference was observed with the mutant genotype (AA) of the rs9939609 variant. These findings were consistent with previous studies that found no relationship between *FTO* and androgen levels in the UK population and no differences in testosterone, SHBG, and FAI across genotypes in the Polish population [18, 33].

The *FTO* protein plays a crucial role in RNA demethylation, which influences gene expression and may regulate genes involved in energy homeostasis [34]. Additionally, it acts as a transcriptional coactivator, vital in the transcriptional regulation of adipogenesis, implying that *FTO* gene may be interested in fat development and maintenance regulation [35]. Those with the rs9939609 risk allele A have higher *FTO* transcripts [36], connected to gene expression impacting glucose homeostasis [37], liver functions [38], inflammatory markers [39], and insulin. Studies indicate that insulin directly affects the production of androgens in polycystic ovary theca cells, and administering insulin can lead to elevated levels of LH and GnRH [40]. In PCOS, ovarian characteristics involve more pre-antral follicles and delayed maturation due to altered FSH sensitivity or increased LH activity. Abnormal GnRH secretion and excess LH release in PCOS stimulate theca cells to produce excessive androgens [41]. This is crucial in modulating the hyperandrogenism status and is involved in the ovarian dysfunction of PCOS.

The *FTO* gene variant rs9939609 is an intron variant that may be involved in developing PCOS, either directly or indirectly, through BMI. As per our findings, obesity and insulin resistance may play a role in the pathogenesis of PCOS. Furthermore, it implies that hormone levels, specifically total testosterone, may play a role in developing PCOS. Although further research is necessary to verify these findings, they have important implications for developing new PCOS treatment strategies, especially those targeting modifiable risk factors.

The current study is the first to investigate this association in a Gujarati population of Western India. However, this study’s small sample size of PCOS cases may have limited our ability to detect minor differences between *FTO* genotypes. Furthermore, the differences in allelic frequency among studies may be due to the different genetic backgrounds of various ethnic groups and the study’s statistical power (sample size). To examine the role of *FTO* variants in the development of PCOS, larger sample sizes and subgroup analyses of PCOS women are necessary. Nevertheless, the effect sizes observed in this study are comparable to those reported in other PCOS populations.

## Conclusion

Our findings suggest a notable correlation between the *FTO* gene variant rs9939609 and increased obesity prevalence among women of Gujarati ethnicity. Additionally, significant disparities in insulin resistance were observed between obese and non-obese PCOS patients. This prompts the hypothesis that the *FTO* gene may contribute to PCOS development, possibly via its impact on BMI or obesity. To gain a deeper understanding, further investigation across diverse ethnic groups is necessary to elucidate the intricate connection between *FTO* gene polymorphism, PCOS, and obesity.

### Supplementary Information

**Additional file 1: Supplementary Figure 1.** Recruitment of PCOS samples and healthy controls. **Supplementary Table 1.** Risk factors for PCOS.

## Data Availability

The data used in the current study are available from the corresponding author on reasonable request.

## Abbreviations

PCOS: Polycystic Ovary Syndrome
FTO: Fat Mass and Obesity-Associated Protein
LH: Luteinizing Hormone
FSH: Follicle Stimulating Hormone
PRL: Prolactin
TSH: Thyroid Stimulating Hormone
SHBG: Sex Hormone-Binding Globulin
FAI: Free Androgen Index
HOMA-IR: Homeostasis Model Assessment of Insulin Resistance
F. Insulin: Fasting Insulin
F. Glucose: Fasting Glucose
DHEAS: Dehydroepiandrosterone Sulphate
E2: Estradiol
T: Testosterone
BMI: Body Mass Index
CLIA: Chemiluminescent immunoassay
DNA: Deoxyribose nucleic acid
EDTA: Ethylene diamine tetra acetic acid
SPSS: Statistical Package for social science
HWE: Hardy Weinberg equilibrium
ORs: Odds ratio
Cis: Confidence intervals
SD: Standard deviation
ANOVA: Analysis of variance
Bp: Base pair
PCR: Polymerase chain reaction
ARMS: Amplification refractory mutation system
SNP: Single Nucleotide Polymorphism
