# Supplementary material for: Association of FTO gene variant rs9939609 with polycystic ovary syndrome from Gujarat, India
Source: BMC Med Genomics. 2023 Sep 14;16:216. doi: 10.1186/s12920-023-01654-0 (PMC10500741; doi:10.1186/s12920-023-01654-0)
Supplement: Supplementary file 1 — Additional file 1: Supplementary Figure 1. Recruitment of PCOS samples and healthy controls. Supplementary Table 1. Risk factors for PCOS. [file 12920_2023_1654_MOESM1_ESM.docx]

Supplementary Information


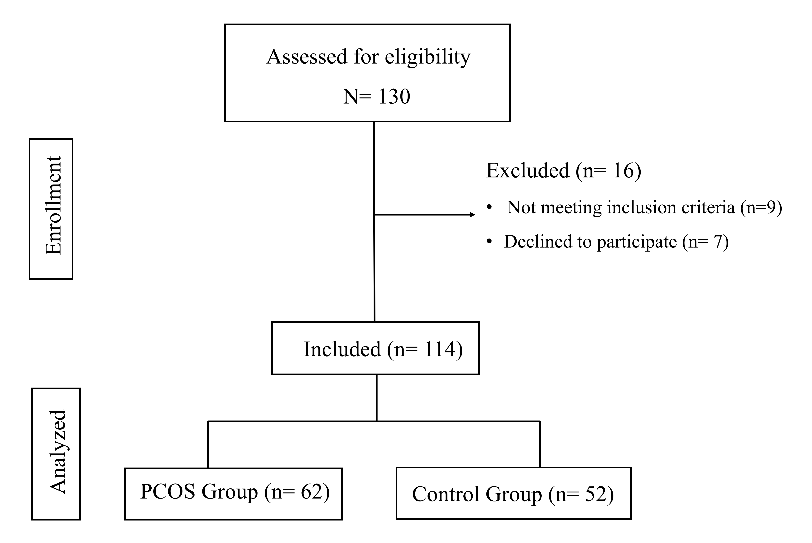


**Supplementary Figure 1:** **Recruitment of PCOS samples and healthy controls.**

**Supplementary Table 1: Risk factors for PCOS**

|  | *PCOS* | *Control* | *p-value* | OR |
| --- | --- | --- | --- | --- |
| BMI | 27.23 ± 5.64 | 23.63 ± 5.31 | **0.036** | 1.117 |
| TSH | 2.22 ± 1.1 | 2.15 ± 1.32 | 0.372 | 0.829 |
| LH | 6.74 ± 4.43 | 5.35 ± 4.37 | 0.796 | 1.023 |
| FSH | 8.89 ± 7.44 | 7.99 ± 6.48 | 0.516 | 1.025 |
| LHFSH | 1.00 ± 0.93 | 0.94 ± 1.06 | 0.357 | 1.371 |
| E2 | 80.2 ± 111.49 | 75.32 ± 25.36 | 0.631 | 0.999 |
| TT | 23.18 ± 13.97 | 16.17 ± 8.89 | **0.004** | 1.131 |
| PRL | 15.68 ± 11.47 | 17.83 ± 22.01 | 0.869 | 0.998 |
| DHEAS | 126.03 ± 58.2 | 199.67 ± 136.53 | **0.001** | 0.982 |
| FTO (TT) | 27 (43.55%) | 21 (40.38) | 0.874 |  |
| FTO (TA) | 12 (19.35) | 16 (30.77) | 0.605 | 0.721 |
| FTO (AA) | 23 (37.10) | 15 (28.85) | 0.860 | 0.902 |

*BMI* Body Mass Index, *TSH* Thyroid stimulating hormone, *LH* luteinizing hormone, *FSH* follicle stimulating hormone, *E2* estradiol, *TT* total testosterone, *PRL* prolactin, *DHEAS* dehydroepiandrosterone sulphate, Logistic Regression analysis*p<0.05 significant.


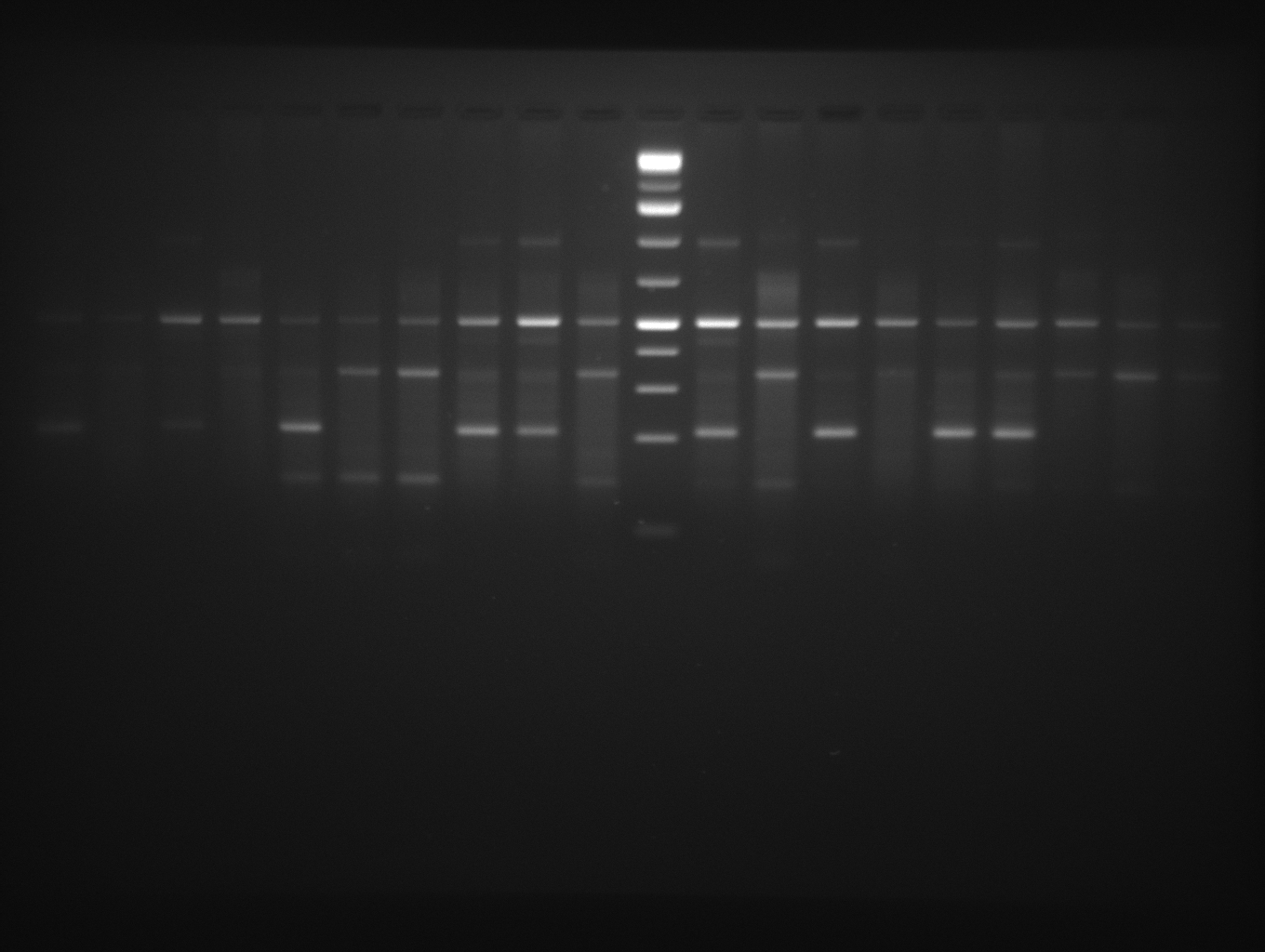


**Figure 1: Genotyping of FTO variant rs9939609 through tetra-ARMS PCR.** Original file is uncropped.
